# Inhibition of glutaminolysis alone and in combination with HDAC inhibitor has anti-myeloma therapeutic effects

**DOI:** 10.20517/cdr.2024.35

**Published:** 2024-06-24

**Authors:** Seiichi Okabe, Yuko Tanaka, Mitsuru Moriyama, Akihiko Gotoh

**Affiliations:** Department of Hematology, Tokyo Medical University, Tokyo 160-0023, Japan.

**Keywords:** Multiple myeloma, glutaminolysis, *GLS1*, HDAC inhibitor

## Abstract

**Aim:** This study aimed to investigate drug candidates and their efficacy in treating refractory multiple myeloma (MM) despite significant therapeutic advances and the introduction of novel agents. Our study focused on how myeloma cells mediate the metabolic pathways essential for survival. Therefore, we examined the role of glutaminolysis in this process.

**Methods:** We investigated the role of glutaminolysis in myeloma cell growth. In addition, we analyzed the ability of CB-839 (telaglenastat), a glutaminase (GLS) inhibitor, to suppress myeloma cell proliferation and enhance the sensitivity to histone deacetylase (HDAC) inhibitors.

**Results:** Glutamate deprivation significantly reduced MM cell proliferation. We observed an upregulation of GLS1 expression in MM cell lines compared to that in normal controls. CB-839 inhibits MM cell proliferation in a dose-dependent manner, resulting in enhanced cytotoxicity. Additionally, intracellular α-ketoglutarate and nicotinamide adenine dinucleotide phosphate levels decreased after CB-839 administration. Combining panobinostat with CB-839 resulted in enhanced cytotoxicity and increased caspase 3/7 activity. Cells transfected with GLS shRNA exhibited reduced cell viability and elevated sub-G1 phase according to cell cycle analysis results. Compared to control cells, these cells also showed increased sensitivity to panobinostat.

**Conclusion:** Glutaminolysis contributes to the viability of MM cells, and the GLS inhibitor CB-839 has been proven to be an effective treatment for enhancing the cytotoxic effect of HDAC inhibition. These results are clinically relevant and suggest that CB-839 is a potential therapeutic candidate for patients with MM.

## INTRODUCTION

Multiple myeloma (MM) is a type of blood cancer characterized by overgrowth of abnormal plasma cells within the bone marrow^[1]^. These clonal plasma cells, which are central to the pathogenesis of MM, originate from the post-germinal center B cells^[2]^. The journey for almost all MM patients begins with an asymptomatic premalignant stage known as monoclonal gammopathy of unknown significance (MGUS)^[3]^. Clinically, the diagnosis of MM requires the presence of end-organ damage as defined by the CRAB criteria, which include elevated calcium levels, anemia, renal insufficiency, and bone lesions^[3]^. Significant progress has been made in MM therapy over the last 25 years because of the introduction of numerous new classes of medicines, including proteasome inhibitors, immunomodulatory medications, and monoclonal antibodies^[4]^. Despite therapeutic advancements, high-risk diseases continue to exhibit poor outcomes^[5]^. Furthermore, in many patients with MM, the emergence of resistance mechanisms contributes to relapse. Consequently, the clinical management of patients with MM to improve overall survival remains a challenge.

Warburg *et al.* revealed that proliferating cancer cells prefer to convert glucose to lactate, even in the presence of oxygen, instead of redirecting pyruvate to the tricarboxylic acid cycle^[6]^. This phenomenon is termed aerobic glycolysis, or the Warburg effect^[7]^. Glutamine (Gln) and glucose are pivotal for meeting bioenergetic and biosynthetic demands^[8]^. Gln, an abundant circulating amino acid in blood and muscles, is an essential resource for energy production^[9]^. Furthermore, Gln is crucial for numerous fundamental biological activities of cancer cells^[8]^. Cancer cells undergo metabolic reprogramming, which renders them highly dependent on Gln for survival and rapid proliferation^[8]^. Gln enters cells and is a versatile precursor for the synthesis of various amino acids, proteins, nucleotides, and other molecules that are crucial for biological activity. Furthermore, Gln contributes to maintaining redox equilibrium by delivering essential molecules such as glutathione and dihydro-nicotinamide-adenine-dinucleotide phosphate (NADPH)^[10]^. Glutaminase (GLS), a tissue-specific isoenzyme, functions as an amidohydrolase that catalyzes the conversion of Gln to glutamate. It was the first enzyme involved in Gln metabolism with two paralogous *GLS* genes: *GLS1* (or *GLS*) and *GLS2*^[11]^.

Several studies have investigated the potential of GLS inhibitors in various types^[12]^. CB-839, also known as telaglenastat, is a potent and non-competitive allosteric GLS inhibitor. This study aimed to unveil the regulatory mechanisms underlying the role of Gln, which fuels the Warburg effect and eventually drives MM cell proliferation. This study aimed to identify the potential treatment options for MM based on Gln metabolism.

Panobinostat is an orally administered histone deacetylase (HDAC) inhibitor that suppresses the enzymatic activity of HDAC proteins in the nanomolar range^[13]^. HDAC facilitates the catalytic removal of acetyl groups from lysine residues of histones and non-histone proteins^[14]^. Karagiannis *et al.* reported that inhibition of HDACs significantly affects cancer cells by causing comprehensive modifications in chromatin, altering metabolic gene expression, and restricting the activity of amino acid and nucleotide metabolic pathways, ultimately leading to reduced cell proliferation^[15]^. Therefore, considering the clinical use of HDAC inhibitors, including panobinostat, in patients with MM, we sought to determine whether combining panobinostat with CB-839 could enhance cytotoxicity in myeloma cells.

## METHODS

### Reagents

The GLS inhibitor CB-839 (telaglenastat) and the pan-HDAC inhibitor panobinostat (LBH589) were obtained from Selleck Chemicals (Houston, TX, USA). All chemicals were dissolved in dimethyl sulfoxide. All other reagents were purchased from Merck KGaA (Darmstadt, Germany).

### Cell lines, cell culture and primary samples

The MM cell lines U266, RPMI 8226, MM.1S, and MM.1R were purchased from American Type Culture Collection (ATCC, Manassas, VA, USA). Additionally, the Japanese Collection of Research Bioresources Cell Bank (located in Ibaraki, Osaka, Japan) supplied the bortezomib-resistant myeloma cell line KMS-11/bortezomib (BTZ). Previous studies have documented the characteristics of KMS-11/BTZ cells^[16]^. MM cell lines were maintained under standard conditions at 37 °C in a humidified atmosphere in Roswell Park Memorial Institute 1640 (RPMI 1640) media supplemented with 2 mmol/L Gln and 10% fetal bovine serum (FBS). Gln depletion experiments involved the cultivation of MM cells in Gln-free RPMI 1640 medium. After obtaining written informed consent, peripheral blood mononuclear cells (PBMCs) were isolated from blood samples of patients with plasma cell leukemia and healthy volunteers using Lymphocyte Separation Medium 1077 (PromoCell GmbH, Heidelberg, Germany). Following isolation, PBMCs were cultured in a medium containing 20% FBS at a density of 5 × 10^5^ cells/mL, while the others were used for polymerase chain reaction (PCR) assays. This study was approved by the Ethics Committee of the Tokyo Medical University (approval number: T2023-0105).

### Data collection and processing

We retrieved microarray data, specifically GSE80608, comprising 10 normal bone marrow (BM) samples, 10 BM samples from MGUS, and 10 BM samples of mesenchymal stem cells (MSC) from the iliac crest of 10 patients with myeloma, from the Gene Expression Omnibus (GEO) database (https://www.ncbi.nlm.nih.gov/geo/)^[17]^. Data analysis was performed using the GEO2R website, which is an interactive tool for comparing various sample groups in a GEO series. The data were downloaded in the SOFT format, converted to XLS files, and analyzed using Microsoft Office Excel (Microsoft Corporation, Redmond, WA, USA). Differentially expressed genes (DEGs) were identified based on an adjusted *P* value (< 0.05) and a log2 fold change (≥ 1.0 or ≤ -1.0).

### Cell viability assay

MM cells were cultured at a density of 2 × 10^5^ cells/mL and treated with HDAC inhibitors panobinostat and CB-839 for 72 h. Subsequently, cell viability was assessed using the Cell Counting Kit-8 (Dojindo Laboratories, Mashikimachi, Kumamoto, Japan) according to the manufacturer’s instructions. Cell Counting Kit-8 measures dehydrogenase activity in living cells by utilizing intracellular metabolic activity with nicotinamide adenine dinucleotide (NADH) as an indicator. The 2-[2-methoxy-4-nitrophenyl]-3-[4-nitrophenyl]-5-[2,4-disulfophenyl]-2H-tetrazolium monosodium salt (WST-8) produces a water-soluble formazan dye upon reduction in the presence of an electron mediator. Absorbance was recorded at 450 nm using an EnSpire Multimode Plate Reader (PerkinElmer, Waltham, MA, USA). Gln deprivation experiments were performed using Gln-free RPMI 1640 medium. In this experiment, additional glucose was added to achieve a concentration of 4,500 mg/L.

### Caspase 3/7 activity

Caspase activity was assessed with the Caspase-Glo® 3/7 assay kit by Promega, following the recommended protocols provided by the manufacturer. After incubation for 48 h with the specified concentrations of panobinostat and/or CB-839, the luminescence of each sample was quantified using EnSpire.

### Cytotoxicity assay

Cytotoxicity assessments were conducted in MM cells treated with defined concentrations of panobinostat and/or CB-839 for 72 h. Cytotoxicity was evaluated using the Cytotoxicity lactate dehydrogenase (LDH) Assay Kit (Dojindo Laboratories, Kumamoto, Japan). The LDH Assay Kit quantifies the activity of LDH, which is released as a result of cell membrane damage and serves as an indicator of cell death. The absorbance at 490 nm, which is a measure of the amount of LDH released from dead cells, was measured using a Plate Reader.

### Quantitative real-time reverse transcription-polymerase chain reaction analysis

Total RNA was extracted from MM cells or PBMCs using the RNAqueous®-4PCR Kit (Life Technologies Japan KK, Minato-ku, Tokyo, Japan). Reverse transcription was performed using a First-Strand cDNA Synthesis Kit (OriGene Technologies, Rockville, MD, USA). Quantitative real-time reverse transcription-polymerase chain reaction (RT-PCR) was conducted using the Roche Light Cycler 2.0 detection system (Roche Diagnostic Gmbh, Minato-ku, Tokyo, Japan). Specific primers for *GLS1*, *GLS2* and actin beta (*ACTB*) were procured from Takara Bio Inc. (Otsu, Shiga, Japan). Specific gene expression was quantified using a SYBR Green PCR Kit (Roche) according to the manufacturer’s guidelines.

### Short-hairpin RNA transfection

A lentiviral vector designed for mammalian GLS1 expression was used to transfect short hairpin RNAs (shRNAs). The control shRNA vector was obtained from Vector Builder Japan, Inc. (Yokohama, Kagawa, Japan). MM cells were grown in a 6-well culture dish and were cultured in medium supplemented with 8 µg/mL of polybrene (hexadimethrine bromide) (Merck KGaA) for 24 h. Cells were infected with lentiviral vectors according to the manufacturer’s instructions. The medium was replaced after a 24-h incubation period, and *GLS* expression was assessed by RT-PCR and immunoblotting.

### Adenosine triphosphate assays

The U266 cells were exposed to specific concentrations of CB-839 for 24 h. In certain experiments, cells were cultured in Gln-free RPMI 1640 medium. Intracellular adenosine triphosphate (ATP) concentrations were determined using the Cell ATP Test Reagent Kit Version 2 [TOYO B-Net (Tokyo, Japan)] following the manufacturer’s protocol. Luciferase activity was quantified using the EnSpire Multimode Plate Reader (PerkinElmer).

### Enzyme-linked immunosorbent assays

U266 cells were cultured in medium containing a specified concentration of CB-839. After 24 h, the U266 cells were harvested and stored at -80 °C. NADPH levels were analyzed using the NADP/NADPH Assay Kit-WST (Dojindo Laboratories) following the manufacturer’s protocol. The intracellular NADPH concentration was precisely measured by heat-induced treatment of the cell lysate with the extraction buffer provided in the kit. Intracellular nicotinamide-adenine-dinucleotide phosphate (NADP+) levels were determined by subtracting the quantity of NADPH from the total NADP+/NADPH ratio. The absorbance was measured at 450 nm using an EnSpire Multimode Plate Reader. Intracellular α-ketoglutarate (α-KG) was quantified using the α-Ketoglutarate Assay Kit (Sigma-Aldrich, Inc., St. Louis, MO, USA), adhering to the manufacturer’s protocol. After 24 h of incubation with the specified concentration of CB-839, the α-KG concentration was determined using a coupled enzyme assay, yielding a colorimetric readout at 570 nm. Histone acetylation was assessed using the CycLex® Cellular Histone Acetylation Assay Kit (MBL, Nagoya, Aichi, Japan) following the manufacturer’s protocols. The absorbance was recorded at 450 and 550 nm in each well.

### Cell cycle analysis

Cell cycle analysis was performed using the BD CycleTest Plus DNA Reagent Kit (Becton-Dickinson, Mountain View, CA, USA) following the manufacturer’s protocol. U266 cells were cultured for 24 h with specified concentrations of panobinostat and/or CB-839. The analysis of DNA content distribution was conducted using the BD FACSVers^TM^ Flow Cytometer and BD FACSuite^TM^ software, both provided by Becton-Dickinson.

### Immunoblotting

Immunoblotting was performed according to previously established procedures^[18,19]^. U266 cells were treated with various concentrations of panobinostat and/or CB-839 for 24 h. The shRNA-transfected U266 cells were collected and lysed using a radioimmunoprecipitation assay buffer. Equal amounts of protein mixed with 2× Laemmli sample buffer were applied to mini-protein TGX gels (Bio-Rad, Hercules, CA, USA). The cell lysates were transferred to polyvinylidene difluoride membranes (Millipore, Billerica, MA, USA). After blocking, the membranes were incubated with primary antibodies at room temperature for 1 h. The primary antibodies included cleaved caspase 3: Cell Signaling Technology (Danvers, MA, USA), cleaved poly (ADP-ribose) polymerase (PARP): Cell Signaling Technology, acetyl Histone H4: Merck KGaA, GLS (Abcam; Cambridge, UK), and β-actin (Santa Cruz Biotechnology, Santa Cruz, CA, USA). The membranes were subsequently incubated with secondary antibodies (Cell Signaling Technology) and visualized using an enhanced chemiluminescence system (Amersham Pharmacia Biotech (Little Chalfont, UK).

### Statistical analyses

All data presentation and analyses were conducted using the Prism 10 software (GraphPad Software, San Diego, CA, USA). Statistical significance was evaluated using a two-tailed Student’s *t*-test. Data are presented as the means ± SDs and subjected to a one-way analysis of variance, followed by Tukey’s or Dunnett’s post hoc tests for multiple comparisons or a two-tailed t-test for comparing two groups. Significance levels were denoted as ^*^P < 0.05, ^**^P < 0.01, ^***^P < 0.001, and ^****^P < 0.0001.

## RESULTS

### Analysis of Gln in myeloma cell lines and gene expression in myeloma samples

**Figure 1:**
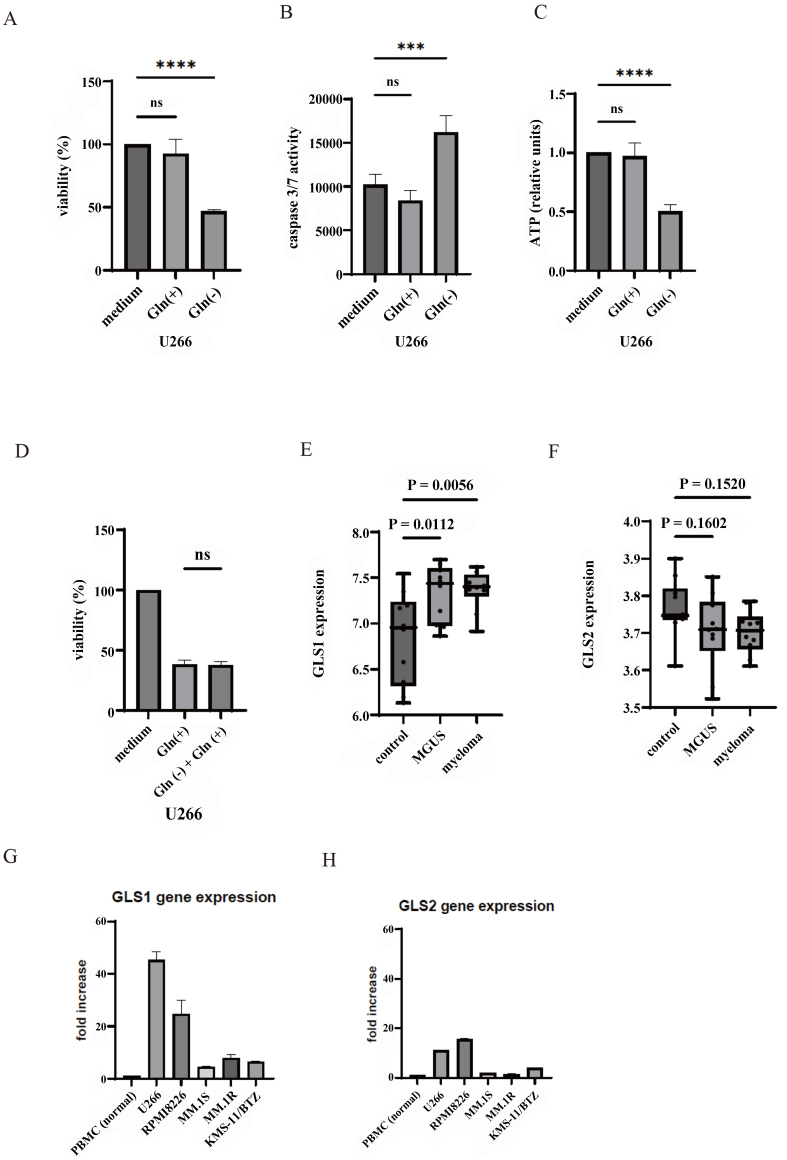
Glutaminolysis in U266 cells and *GLS1* and *GLS2* expression in monoclonal gammopathy of undetermined significance and MM cells. (A) The viability of cells was evaluated by culturing them in medium with or without Gln for 72 h; (B) Caspase 3/7 activity was measured; and (C) intracellular ATP levels were quantified. Statistical significance is denoted as ^***^P < 0.001 and ^****^P < 0.0001 compared to the complete medium cultured cells. ns: not significant; (D) U266 cells were cultured in RPMI 1640 medium, without Gln or without Gln plus glucose, for 72 h. Cell viability was analyzed. ns: not significant; (E and F) *GLS1* and *GLS2* expression levels were validated using data from the GEO database. Statistical significance compared to normal is indicated. ns: not significant. (G and H) Total RNA was extracted from normal PBMC and myeloma cell lines. *GLS1* and *GLS2* gene expressions were analyzed using RT-PCR. GLS: Glutaminase; MM: multiple myeloma; ATP: adenosine triphosphate; RPMI 1640: Roswell Park Memorial Institute 1640; GEO: Gene Expression Omnibus; PBMC: peripheral blood mononuclear cell; RT-PCR: reverse transcription-polymerase chain reaction.

Gln, the most abundant free amino acid in plasma, is pivotal for various metabolic processes^[8]^. First, we investigated the correlation between Gln expression and proliferation in the U266 myeloma cell line. The removal of Gln from RPMI 1640 medium reduced cell proliferation and increased caspase 3/7 activity [Figure 1A and B]. Gln-driven oxidative phosphorylation is the primary source of ATP. Consequently, we assessed the intracellular ATP levels in U266 cells, which revealed a decrease in Gln removal from the medium [Figure 1C]. The addition of glucose to the medium without Gln did not affect the viability of U266 cells [Figure 1D].

Additionally, we explored the gene expression patterns of glutaminases (*GLS1* and *GLS2*) using publicly available functional genomic data. The study GSE80608 comprised 10 normal bone marrow samples, 10 from patients with MGUS, and 10 from the iliac crest of individuals diagnosed with myeloma of MSC^[17]^. Data from the Gene Expression Omnibus database (National Center for Biotechnology Information, Bethesda, MD, USA) showed an elevation in *GLS1* expression in MGUS and MM samples compared to normal control samples [Figure 1E]. In contrast, there were no significant alterations in the gene expression of *GLS2* [Figure 1F]. Collectively, these findings suggested that Gln and *GLS1*, but not *GLS2*, support myeloma cell progression. We analyzed that gene expression of *GLS1* was increased in MM cell lines compared to that in normal samples [Figure 1G]. GLS2 is an enzyme responsible for the metabolism of Gln, which serves as a primary source of energy and a fundamental building block in tumor cells^[20]^. In certain cancers, GLS2 functions in a manner contrary to its role as a tumor suppressor; however, in specific myeloma cell lines, increased expression has been noted [Figure 1H]. This elevation may serve as an adaptive mechanism to metabolic stress, allowing myeloma cells to optimize their energy and nutrient acquisition through improved Gln metabolism; thereby, *GLS2* gene also supports the proliferation and survival of myeloma cell lines.

### CB-839 reduces myeloma cell line growth and NADPH metabolism

**Figure 2:**
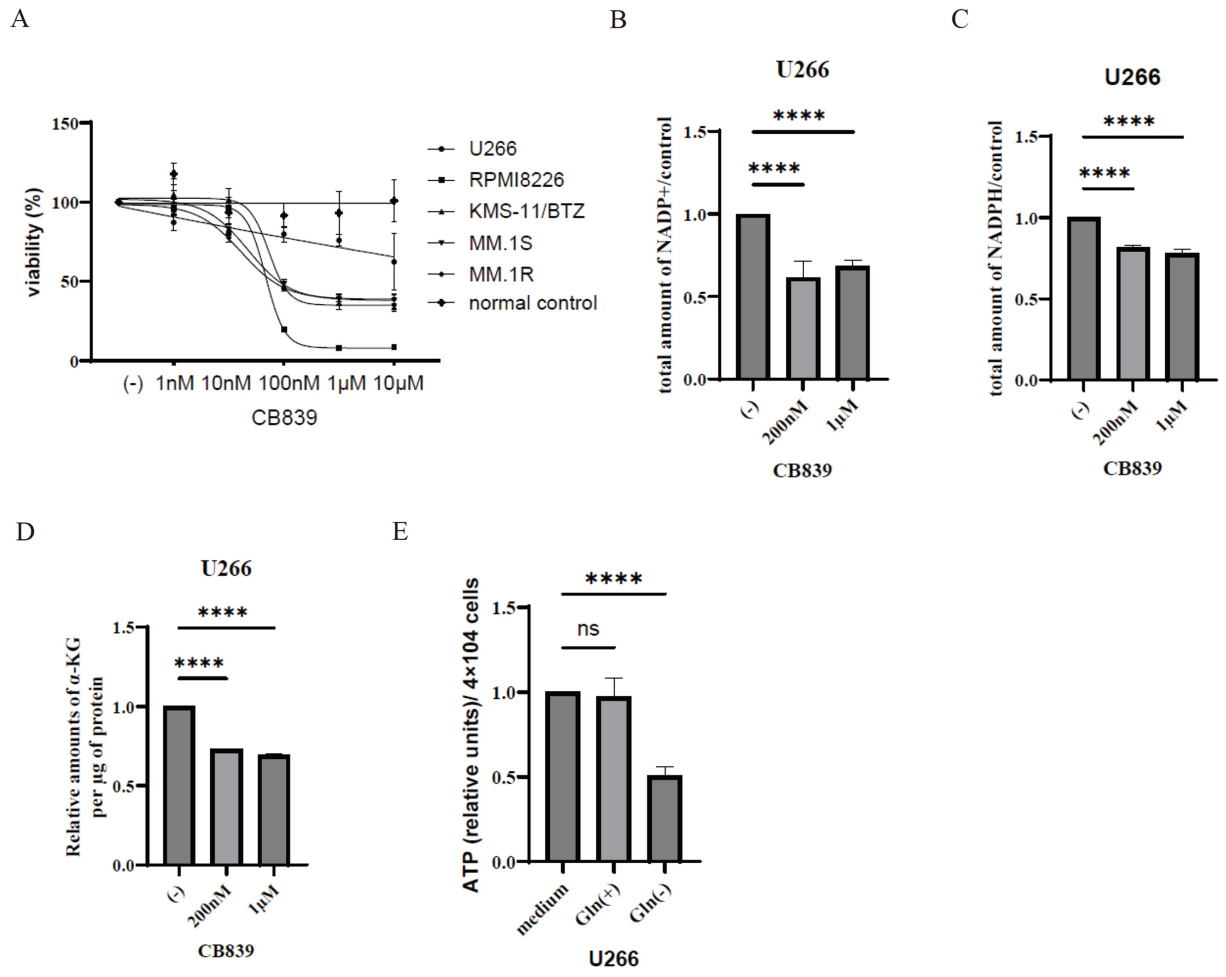
Activity of GLS1 inhibitors in MM cells. (A) MM cell lines and PBMC of normal control samples were cultured with varying concentrations of CB-839. After 72 h, the relative cell counts were assessed; (B-D) U266 cells were treated with the indicated concentrations of CB-839 for 24 h. The total amounts of NADP+ or NADPH and α-KG were analyzed as described in the materials and methods section. Statistical significance is denoted as ^****^P < 0.0001 compared to the control. ns: not significant. (E) U266 cells were treated with the indicated concentrations of CB-839 for 24 h. Intracellular ATP levels in U266 cells (4 × 10^4^) were quantified. Statistical significance is indicated as ^****^P < 0.0001 compared to the control. ns: not significant. GLS: Glutaminase; MM: multiple myeloma; PBMC: peripheral blood mononuclear cell; NADP+: nicotinamide-adenine-dinucleotide phosphate; NADPH: dihydro-nicotinamide-adenine-dinucleotide phosphate; α-KG: α-ketoglutarate; ATP: adenosine triphosphate.

We found that the expression of the *GLS1* gene was higher in the MM cell lines. Considering this finding, we conducted a study to assess the influence of CB-839, an oral medication that potently and specifically inhibits GLS, and analyzed its effect on myeloma cell activity. Our results demonstrate that CB-839 effectively inhibits the viability of all tested myeloma cell lines. This inhibition is likely due to reduced glutamate production, which leads to metabolic stress, consequently inhibiting cell viability and inducing cell death in myeloma cells (inhibitory concentration curve) [Figure 2A]. In contrast, the PBMCs from healthy volunteers were unaffected by CB-839, indicating a selective cytotoxic effect on myeloma cells. Previous studies have demonstrated that lactate and Gln support NADPH production^[21]^. *GLS1* is important for MM cell metabolism and survival, and is involved in plasmacytoma pathogenesis^[22]^. Therefore, we investigated the NADPH metabolism following CB-839 treatment. The total levels of both NADP+ and NADPH were significantly reduced in response to CB-839 treatment in U266 cells [Figure 2B and C], suggesting that the levels of NADP and NADPH were affected by GLS activity through its role in cellular metabolism. Notably, extracellular Gln traverses the plasma membrane and undergoes conversion into α-KG through the GLS1 and GLS2 pathways. In this context, CB-839 induced a dose-dependent reduction in the levels of α-KG in U266 cells [Figure 2D]. Furthermore, we measured intracellular ATP levels in 4 × 10^4^ U266 cells, revealing a decrease in intracellular ATP in response to CB-839 treatment in U266 cells [Figure 2E]. By inhibiting glutaminolysis, CB839 reduces the number of substrates available for ATP production, resulting in reduced intracellular ATP levels. Therefore, the possibility that CB839-induced cell death is associated with decreased ATP production cannot be ruled out. Nevertheless, this metabolic disruption may play a role in the observed inhibition of cell proliferation and the induction of cell death following CB839 treatment.

### Panobinostat inhibits myeloma cell proliferation

**Figure 3:**
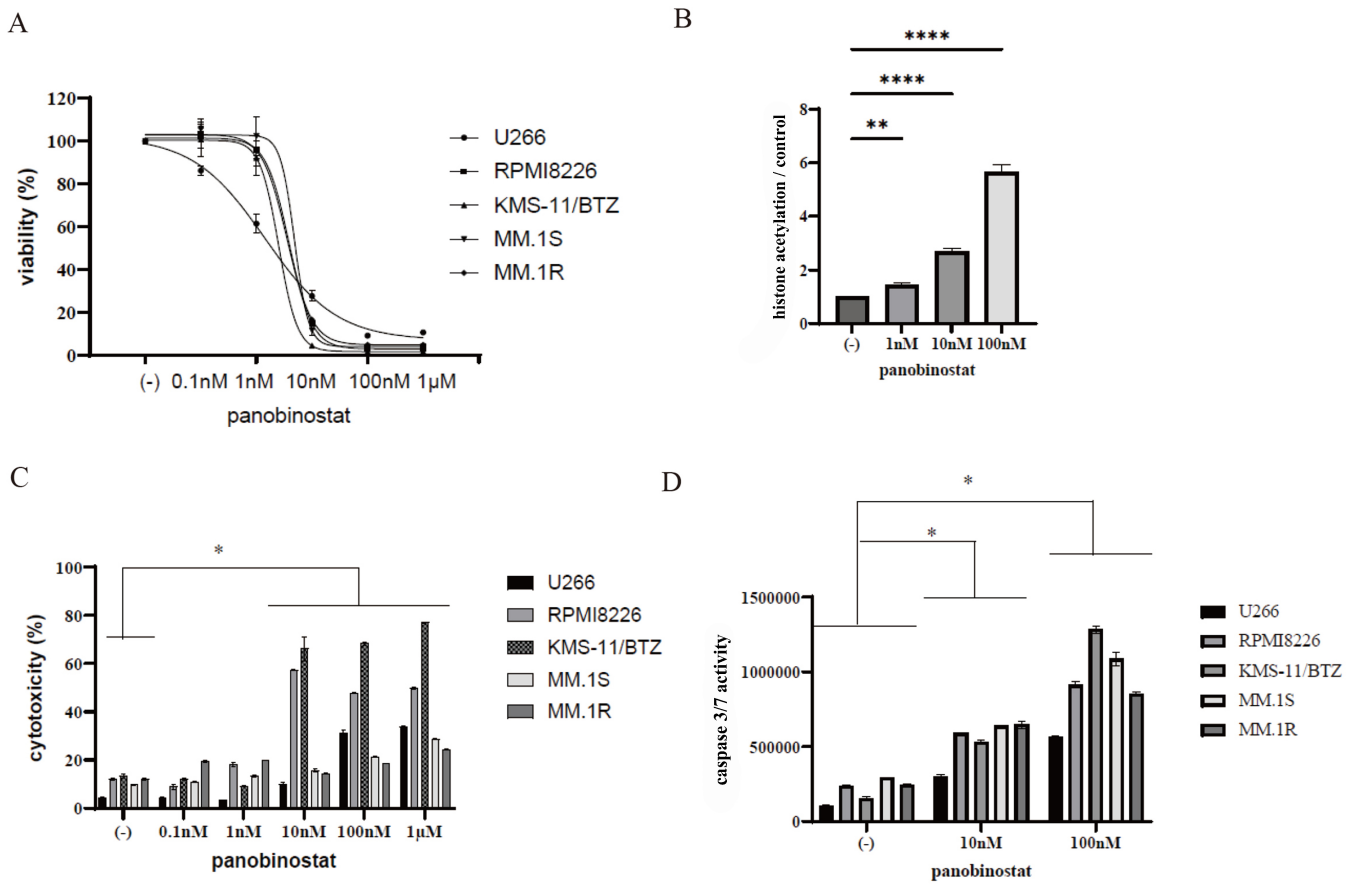
Activity of HDAC inhibitors in MM cells. (A) To assess the effects of panobinostat on MM cells, the cells were cultured for 72 h with different concentrations of the drug, and then the relative cell counts were evaluated. (B) U266 cells were treated with panobinostat for 24 h, and histone acetylation was evaluated. Statistical significance is denoted as ^**^P < 0.01 and ^****^P < 0.0001 compared to the control. (C and D) MM cells were incubated with different concentrations of panobinostat for 48 or 72 h. Subsequently, cytotoxicity (C) and caspase 3/7 activity (D) were measured. Statistical significance is denoted as ^*^P < 0.05 compared to the control. HDAC: Histone deacetylase; MM: multiple myeloma.

HDAC inhibitors are epigenetic regulators of cellular metabolism^[15]^. Interactions between metabolism and histone acetylation affect several biological processes. Panobinostat is an oral pan-deacetylase inhibitor that acts against myeloma by modulating gene expression and disrupting protein metabolism^[23]^. Panobinostat showed synergistic effects on myeloma cells when used in combination with bortezomib and dexamethasone, especially in patients with refractory myeloma^[23]^. Therefore, in this study, we investigated the activity of panobinostat. Figure 3A shows a dose-response curve based on the relative myeloma cell count normalized to that of the untreated control (inhibitory concentration curve). Our results revealed that panobinostat effectively reduced cell viability and induced cell death in all the myeloma cell lines [Figure 3A]. Histone deacetylases (HDACs) play pivotal roles in regulating histone acetylation. Treatment with panobinostat led to dose-dependent accumulation of acetylated histones in U266 cells [Figure 3B]. In this study, we measured the number of viable or dead cells using two distinct methodologies: the Cell Counting Kit-8 assay, which quantifies intracellular metabolic activity by utilizing NADH as an indicator to verify cell viability, and the Cytotoxicity LDH Assay Kit-WST, which quantifies free LDH resulting from cellular membrane damage. To further assess its effect on cell viability, we conducted a cytotoxicity analysis to determine the percentage of dead cells. Incubation of myeloma cell lines with various concentrations of panobinostat for 72 h increased the cytotoxicity [Figure 3C]. Owing to variations in the indicators, the results in cell lines may be discrepant. Additionally, caspase 3/7 activity was enhanced upon panobinostat treatment [Figure 3D].

### Combination treatment with panobinostat and CB-839 inhibited myeloma cell growth

**Figure 4:**
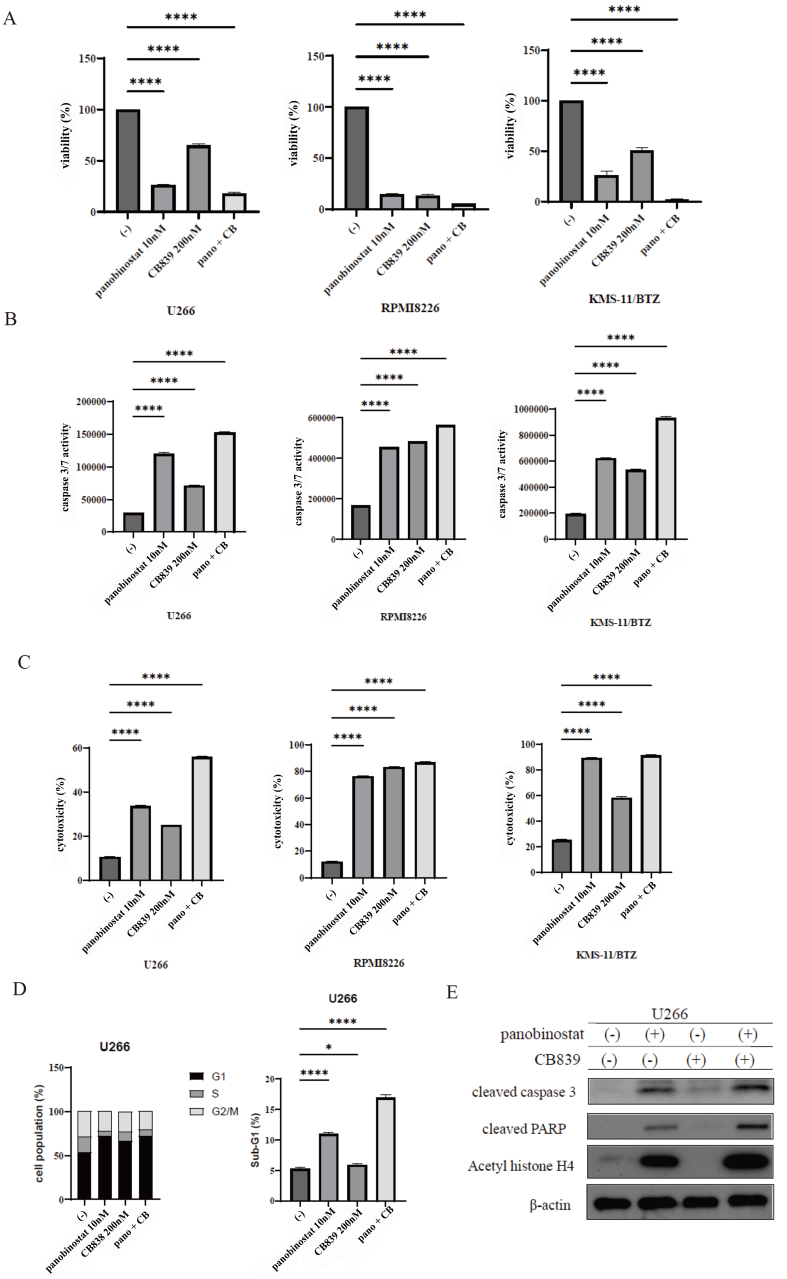
Cotreatment with panobinostat and CB-839 induced cytotoxicity in MM cells. (A-C) U266, RPMI 8226, and KMS-11/BTZ cells were cultured with panobinostat and/or CB-839 for 48 or 72 h. (A) Cell proliferation, (B) caspase 3/7 activity, and (C) cytotoxicity were evaluated. Statistical significance is denoted as ^****^P < 0.0001 compared to the control. The experiment was repeated three times. (D) Cell cycle profiling was determined as described in the materials and method. U266 cells were treated with panobinostat (10 nM) and/or CB-839 (200 nM) for 24 h. A representative histogram for each treatment condition is illustrated. ^*^P < 0.05 and ^****^P < 0.0001 compared to the control. The experiment was conducted three times; (E) U266 cell lines were treated with panobinostat (10 nM) and/or CB-839 (200 nM). Total protein extracts were analyzed using immunoblotting against cleaved caspase 3, cleaved PARP, acetyl histone H4, and β-actin antibodies. MM: Multiple myeloma; RPMI: Roswell Park Memorial Institute; BTZ: bortezomib; PARP: poly (ADP-ribose) polymerase.

Combining the HDAC inhibitors, panobinostat and CB-839, may be a valuable approach for treating myeloma. By targeting different metabolic pathways and epigenetic mechanisms, this combination therapy may be an effective means of treating diseases. Therefore, we extended our investigation to explore the effects of the drug combinations on MM cells. MM cells were treated with panobinostat and/or CB-839 for 72 h. Our findings demonstrate that panobinostat and CB-839 therapy significantly inhibited the proliferation of myeloma cells, including bortezomib-resistant cells [Figure 4A]. The Chou-Talalay method is frequently used to assess synergistic effects of drug combinations^[24]^. A Combination Index (CI) is used to measure the extent of interactions. Our findings showed that the CI was < 1, which suggested that the drug combination had synergistic effects. Caspase 3/7 activity and cytotoxicity were also enhanced by combination treatment [Figure 4B and C]. As the growth of myeloma cells decreased, we performed cell cycle analysis using flow cytometry. Panobinostat induced G1 cell cycle arrest, and the combined treatment with panobinostat and CB-839 increased the sub-G1 cell population [Figure 4D]. Immunoblot analysis revealed that cleaved caspase 3 and cleaved PARP levels were elevated by the combined treatment with panobinostat and CB-839, and acetylated histone H4 was detected following panobinostat treatment in U266 cells [Figure 4E].

### Knockdown of *GLS1* increased panobinostat sensitivity in myeloma cells, as observed in an analysis of a primary sample

**Figure 5:**
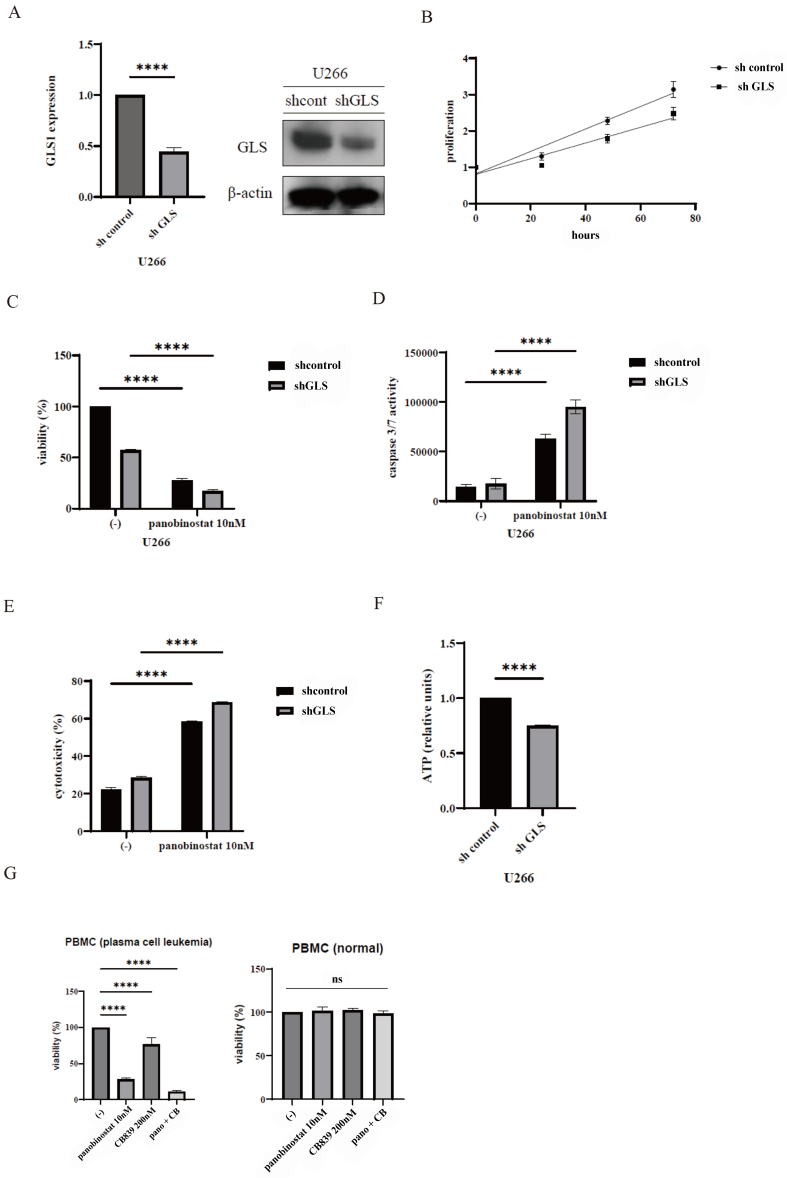
*GLS1* knockdown increased panobinostat activity. (A) Gene expression of GLS1 was analyzed using RT-PCR, and protein expression was examined through immunoblotting using antibodies against GLS1 and β-actin. Statistical significance is indicated as ^****^P < 0.0001 compared to control shRNA-transfected cells. (B) Viability of shRNA-transfected U266 cells was assessed. (C-E) shRNA-transfected U266 cells were incubated with panobinostat. (C) Cell viability, (D) caspase 3/7 activity, and (E) cytotoxicity were evaluated. Statistical significance is denoted as ^****^P < 0.0001 compared with untreated cells. (F) Intracellular ATP levels in shRNA-transfected U266 cells. Statistical significance is denoted as ^****^P < 0.0001 compared with mock-transfected cells. (G) PBMC from leukemia and normal plasma samples were treated with panobinostat and/or CB-839 for 72 h, and cell viability was assessed. ^****^P < 0.0001 compared to control. ns: not significant. The experiments were conducted twice. GLS: Glutaminase; RT-PCR: reverse transcription-polymerase chain reaction; shRNA: short hairpin RNA; ATP: adenosine triphosphate; PBMC: peripheral blood mononuclear cell.

Given the pivotal role of GLS1, we hypothesized that GLS1 might be a critical factor in the survival of MM cells. To analyze the function of GLS1, we used shRNA-mediated gene silencing, a commonly used method to achieve stable gene knockdown. We examined the effects of *GLS1* knockdown in myeloma cells. U266 cells were stably transfected with shRNA expression vectors targeting GLS1 (shGLS1) or with non-targeting shRNAs using a conventional lentiviral construct. The efficiency of gene silencing was evaluated using RT-PCR [Figure 5A]. Immunoblotting confirmed the decrease in *GLS* expression. The cells were cultured at a concentration of 2 × 10^5^ cells/mL and cell proliferation was compared. Notably, cell proliferation was significantly reduced in GLS1 shRNA-transfected U266 cells compared to control shRNA-transfected cells [Figure 5B]. Subsequently, we assessed the efficacy of panobinostat in shRNA-transfected myeloma cells. In GLS1 shRNA-transfected U266 cells, cell viability was reduced in response to panobinostat treatment [Figure 5C]. Caspase 3/7 activity and cytotoxicity increased following panobinostat treatment [Figure 5D and E]. Intracellular ATP levels also decreased [Figure 5F]. Our results suggest that GLS1 knockdown improves the effectiveness of panobinostat and decreases intracellular ATP levels. In subsequent experiments, we used primary samples to investigate the effects of the drug combinations. The combination of panobinostat and CB-839 inhibited the proliferation of PBMCs obtained from patients with plasma cell leukemia [Figure 5G]. In contrast, the analysis of PBMC from normal volunteers indicated that cotreatment with panobinostat and CB-839 did not inhibit cell viability.

## DISCUSSION

Tumor cells, including those in MM, exhibit altered metabolic pathways that are tightly regulated by oncogenic mutations and loss of tumor suppressors^[25]^. A growing body of evidence underscores the crucial function of altered Gln metabolism in tumor cells, as it supports the production of macromolecules that contribute to cancer cell proliferation and survival^[8]^. Our study aligns with these findings, demonstrating the overexpression of the *GLS* gene in patients with MM, as evidenced by the GEO database.

Our results demonstrated that GLS expression is elevated in MM cell lines compared with normal cells. As CB-839 did not affect normal cells, GLS may facilitate increased Gln metabolism in MM cells, allowing them to replenish the citric acid cycle and generate essential molecules required for anabolic growth. Cancer stem cells (CSCs) are believed to play a role in tumor recurrence and drug resistance, presenting a significant challenge for cancer treatment. Gln appears to be a substantial component of cancer cell stemness maintenance^[26]^. In the context of MM, MM stem cells (MMSCs) are identified as cells within the malignant tissue that can self-renew and differentiate into the primary lineages of myeloma plasma cells, which make up the neoplasm. Self-renewal refers to cell division without the loss of differentiation potential, at least in some daughter cells^[27]^. Gln metabolism promotes the maintenance of myeloma stem cells. Therefore, the inhibition of Gln metabolism is a promising adjuvant technique for preventing the development of resistance to standard myeloma therapies and the persistence of MMSCs. In MM cells, Gln dependence is a prominent feature, particularly for bortezomib resistance. Previous studies have shown that targeting coenzyme Q10 production via the mevalonate pathway can synergize with the proteasome inhibitor BTZ^[28]^. Our study extended these findings by demonstrating that GLS inhibition effectively overcomes bortezomib resistance in KMS-11/BTZ cells. This highlights the potential of GLS1 inhibition in MM cells to mitigate proteasome resistance and alter the biosynthetic pathways associated with MM drug resistance.

GLS2 is also involved in the metabolism of Gln, which is a crucial energy source for tumor cells. In certain cancers, GLS2 is overexpressed and contributes to malignancy; however, in other cancers, it serves as a tumor suppressor. Given that GLS2 expression was not elevated in myeloma samples, *GLS2* may act as a tumor suppressor in this context. Furthermore, HDAC inhibitors, such as vorinostat and panobinostat, represent a novel family of medications that target epigenetic gene regulatory enzymes^[29]^. Panobinostat, a pan-HDAC inhibitor, has the potential to improve the prognosis of patients with relapsed or refractory MM when combined with proteasome inhibitors and dexamethasone^[23]^. Our study aligns with these findings and underscores the effectiveness of cotreatment with panobinostat and CB-839 in inducing potent MM cell death compared to each drug administered alone.

Mitochondrial respiration in MM cells is predominantly driven by Gln, suggesting that targeting Gln metabolism may be a strategic approach for MM treatment. Previously, the tolerability of CB-839 as a monotherapy was assessed in a phase 1 dose-escalation trial involving patients with advanced myeloma and was found to be well tolerated^[30]^. Although the primary objectives of this study were safety and tolerability, the preliminary efficacy of a single medication was limited, resulting mostly in long-term stable disease. These findings suggest that CB-839 may be most effective when combined with other anti-MM medications such as panobinostat.

Recent research has shown that CB-839 and EGCG work together to suppress cell growth and promote apoptosis in KM3/BTZ cells, likely by disrupting Gln metabolism and glycolysis^[31]^. The inhibition of glutaminolysis has the potential to trigger apoptosis and could be a valuable therapeutic target for the treatment of MM by promoting MYC degradation^[32]^. Following a single 20-mg oral dose, panobinostat was rapidly absorbed at a peak concentration of 45 nM after 2 h^[33]^. CB-839 has demonstrated a favorable safety and pharmacokinetic/pharmacodynamic (PK/PD) profile, leading to a recommended dose of 800 mg twice daily, achieving a maximum concentration of 3 µM in clinical trials^[34]^. Therefore, drug concentrations have been determined in clinical trials. The administration of panobinostat and CB839 decreased the plasma cell leukemia samples without affecting normal controls and may be a treatment option for refractory myeloma.

A recent report indicated that an ongoing phase 1 study aims to identify the recommended expansion dose and assess the preliminary efficacy of MLN0128 and CB-839 in treating lung squamous cell carcinoma and KRAS-mutant lung adenocarcinoma^[35]^. Additionally, preclinical and clinical trial data have shown that the combination of CB-839 and capecitabine can effectively treat PIK3CA-mutant colorectal cancers^[36]^. Collectively, these observations highlight the potential of combination therapies involving CB-839 and other drugs to elicit enhanced antitumor responses.

We observed that downregulation or inhibition of GLS1 activity may be associated with altered cellular metabolism and increased sensitivity to specific anticancer therapies, including the HDAC inhibitor panobinostat. We also found that CB-839 induced metabolic stress in myeloma cells, which was characterized by reduced ATP production. Targeting glutaminolysis with CB-839 and combining it with panobinostat in MM may exploit metabolic vulnerabilities and epigenetic dysregulation in cancer cells, leading to anti-myeloma effects. The tumor microenvironment has been implicated in the protection of MM cells against antitumor therapy^[37]^. The myeloma tumor microenvironment is thought to play a role in myeloma growth; however, further investigation is needed to elucidate this relationship in future studies.

MM remains an immense challenge as it is currently an incurable disease, emphasizing the urgent need for continued research aimed at achieving a definitive cure. Given the high consumption of Gln by MM cells, GLS is a pivotal enzyme in the glutaminolysis pathway. Further investigations will likely reveal a more comprehensive understanding of the mechanisms underlying the anti-MM effects of the CB-839/panobinostat combination therapy, potentially leading to the discovery of metabolite indicators that can predict the clinical success of GLS inhibitors in MM. Future studies will play a crucial role in elucidating the effect of GLS inhibitors on MM metabolite profiles.

### Limitations of this research

In this study, we investigated the effects of CB-839 on MM cells *in vitro*. However, *in vitro* experiments may not comprehensively replicate the complex interactions within the tumor microenvironment *in vivo*. Despite the promising findings of *in vitro* experiments involving MM cells, clinical data on the safety and efficacy of CB-839 in patients with myeloma are lacking. Therefore, additional clinical trials are essential to assess the drug effectiveness, pharmacokinetics, and potential side effects in humans.

In conclusion, our findings underscore the critical role of glutaminolysis in myeloma cell proliferation and highlight the efficacy of GLS inhibitors in targeting myeloma cells while enhancing the cytotoxic effects of HDAC inhibitors. Furthermore, our results suggest the clinical relevance of GLS inhibitors as potential drug candidates for the treatment of refractory myeloma.

## DECLARATIONS

### Availability of data and materials

All datasets and materials generated in this study can be provided by the corresponding author upon reasonable request.

### Copyright

© The Author(s) 2024.
